# REtools: A laboratory program for restriction enzyme work: enzyme selection and reaction condition assistance

**DOI:** 10.1186/1471-2105-7-98

**Published:** 2006-02-28

**Authors:** Patrick Martin, Kim E Boulukos, Philippe Pognonec

**Affiliations:** 1 CNRS UMR 6548, Parc Valrose, Université de Nice Sophia Antipolis, Nice, France

## Abstract

**Background:**

Restriction enzymes are one of the everyday tools used in molecular biology. The continuously expanding panel of known restriction enzymes (several thousands) renders their optimal use virtually impossible without computerized assistance. Several manufacturers propose on-line sites that assist scientists in their restriction enzyme work, however, none of these sites meet all the actual needs of laboratory workers, and they do not take into account the enzymes actually present in one's own laboratory.

**Results:**

Using FileMaker Pro, we developed a stand-alone application which can run on both PCs and Macintoshes. We called it REtools, for Restriction Enzyme tools. This program, which references all currently known enzymes (>3500), permits the creation and update of a personalized list of restriction enzymes actually available in one's own laboratory. Upon opening the program, scientists will be presented with a user friendly interface that will direct them to different menus, each one corresponding to different situations that restriction enzyme users commonly encounter. We particularly emphasized the ease of use to make REtools a solution that laboratory members would actually want to use.

**Conclusion:**

REtools, a user friendly and easily customized program to organize any laboratory enzyme stock, brings a software solution that will make restriction enzyme use and reaction condition determination straightforward and efficient. The usually unexplored potential of isoschizomers also becomes accessible to all, since REtools proposes all possible enzymes similar to the one(s) chosen by the user. Finally, many of the commonly overlooked subtleties of restriction enzyme work, such as methylation requirement, unusual reaction conditions, or the number of flanking bases required for cleavage, are automatically provided by REtools.

## Background

Restriction enzymes are incontestably the most fundamental tool used in molecular biology. Over the past 40 years, more than 3500 restriction enzymes have been isolated from hundreds of strains of bacteria. It is the sequence specificity of enzymes which renders them so valuable. However, because of the large number of restriction enzymes now available on the market, it is becoming quite difficult to navigate through an ever increasing choice to find the most suitable enzyme that one actually needs. One of the drawbacks is the excessive simplification now seen in many laboratories when it comes to digesting DNA. Quite often, researchers tend to focus on the most commonly used restriction enzymes found in the majority of polylinkers and are afraid to use the so called "exotic" enzymes which may "not work as well". Frequently, researchers are not aware of the potential of isoschizomers recognizing and digesting the same DNA sequence, once again mainly because of the huge panel of enzymes currently available. Indeed, approximately 330 different DNA motifs are recognized by restriction enzymes, each of them being cut by a "prototype" enzyme. But in addition to that prototype, an average of ten other restriction enzymes recognize the same DNA sequence. This is potentially of great interest, when a site is to be used to insert an "incompatible" fragment. For example, ApaI is a widely known and frequently used restriction enzyme, which cuts the sequence GGGCC|C. But an ApaI-digested fragment is only compatible with another ApaI-digested fragment. However, if one uses Bsp120I or BspOMI (both commercially available and cut G|GGCCC) instead of ApaI, then the obtained extremities can be religated with NotI digested fragments (GC|GGCCGC), without any further molecular tinkering. This is not an isolated situation, since other well known enzyme restriction sites can be cleaved with different isoschizomers, such as KpnI with Asp718I, or SmaI with Cfr9I when a non blunt cut is needed.

Therefore, it is quite difficult to be aware of all the possibilities offered by the multiple restriction enzymes available on the market, and frequently one tends to restrict his/her choice to the most commonly known restriction enzymes. To take advantage of the vast panel of restriction enzymes commercially available, one must spend a lot of time browsing through different manufacturers' catalogs or web sites. Some software have been developed which greatly help molecular biologists, including NEB's "NEBCutter" [1], and their well known and exhaustive REbase [2], and Promega's "Restriction Enzymes Resource" [3]. However, these programs have their limitations. In particular, they do not take into account the enzymes actually available in the user's own laboratory. In addition, NEBCutter requires the user to enter the nucleotide sequence to be digested, and thus does not provide information on restriction enzymes *per se*. REbase is exclusively an encyclopedia-like description of all known restriction enzymes, while Promega's application is oriented toward restriction enzymes and buffers specifically distributed by this manufacturer. Recently, an attractive program called EnzymeX, has been developed [4]. Unfortunately, this program only runs on MacOSX10.2 or later versions, and does not provide any information regarding isoschizomer use.

In order to bypass these limitations, we developed a stand-alone application that permits the researcher to find all the information available on all known restriction enzymes (>3500), as well as their cutting characteristics and inter-compatibilities, in just a few clicks. Importantly, REtools is also a way to monitor one's laboratory panel of restriction enzymes, and all searches in REtools indicate whether the chosen enzymes (or equivalents) are available in the user's own laboratory. The interface has been designed to make its use as straightforward as possible, and no training is required to use REtools. On-line help is available throughout the program in case additional information is needed. REtools was developed using FileMaker Pro. It is freely available to the academic community upon request, and does not request a FileMaker Pro license, since it is distributed as a bound solution.

## Implementation

**Figure 1:**
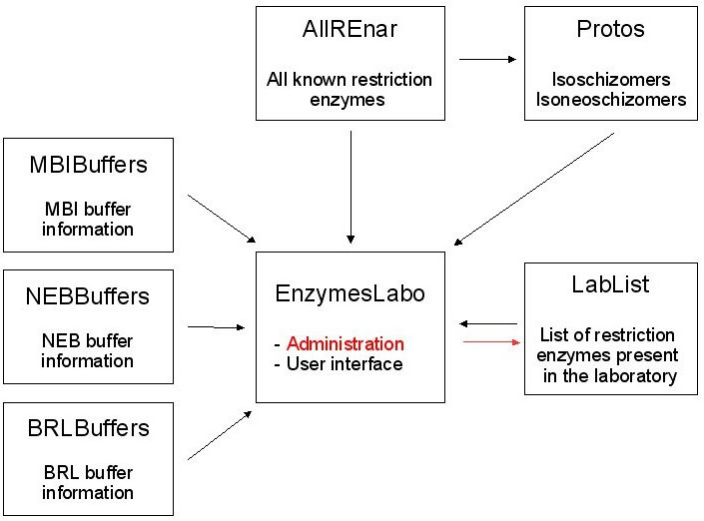
Structure of the REtools relational database. Boxes represent the files constituting REtools. Black arrows indicate data fluxes in response to user requests. The red arrow represents customization of the laboratory enzyme list by the administrator.

REtools, a FileMaker Pro based Runtime solution, is a relational database built around 7 files. The organization of these files are depicted in Fig. 1. The main organizer file called "EnzymesLabo", located in the center, directly handles administration of the lab list as well as user requests. Directly linked to this central file are 6 auxiliary files, containing different data necessary to process users' requests:

1 The "AllREnar" file, which contains all known enzymes as provided by REbase [2], as well their specificities, such as recognition sequence, cleavage, optimal digestion temperature, heat inactivation data, methylation, and minimal number of flanking nucleotides to allow digestion.

2 The "Protos" file contains an exhaustive list of all isoschizomers and neoisoschizomers, taken from the "AllREnar" file.

3 Three files, called "BRLBuffers", "MBIBuffers" and "NEBBuffers", list the recommended buffer for the different restriction enzymes carried by these manufacturers.

4 The "LabList" file is a personalized list which regroups the restriction enzymes present in one's own laboratory.

## Results

REtools, a FileMaker Pro based Runtime solution, is a relational database built around 7 files. The organization of these files are depicted in Fig. 1. The main organizer file called "EnzymesLabo", located in the center, directly handles administration of the lab list as well as user requests. Directly linked to this central file are 6 auxiliary files, containing different data necessary to process users' requests:

1 The "AllREnar" file, which contains all known enzymes as provided by REbase [2], as well their specificities, such as recognition sequence, cleavage, optimal digestion temperature, heat inactivation data, methylation, and minimal number of flanking nucleotides to allow digestion.

2 The "Protos" file contains an exhaustive list of all isoschizomers and neoisoschizomers, taken from the "AllREnar" file.

3 Three files, called "BRLBuffers", "MBIBuffers" and "NEBBuffers", list the recommended buffer for the different restriction enzymes carried by these manufacturers.

4 The "LabList" file is a personalized list which regroups the restriction enzymes present in one's own laboratory.

**Figure 2:**
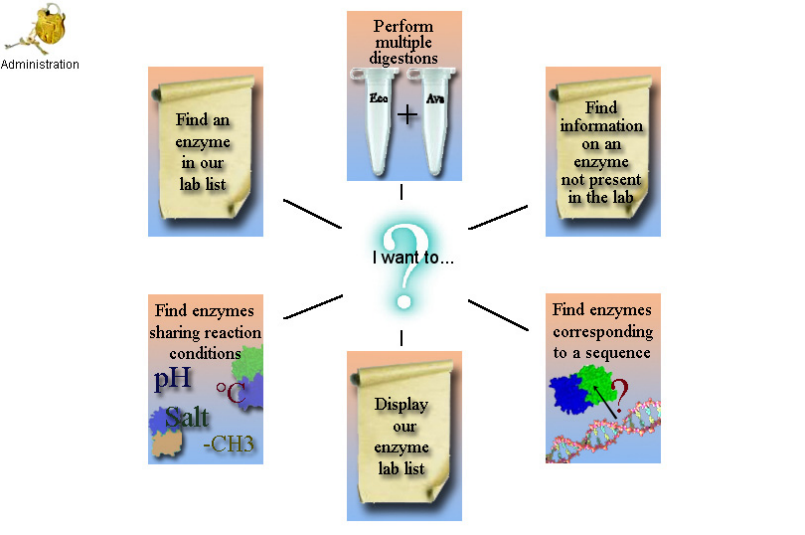
Home page, where the different functions of REtools can be selected.

REtools proposes 7 main functions, directly available from the opening screen (Fig. 2):

- 1. "Administration" (password protected), to customize/update the enzyme list to organize enzymes actually available in one's laboratory.

- 2. "Find an enzyme in our lab list", searches for information on a specific enzyme present in one's laboratory.

- 3. "Perform multiple digestion", searches for optimum conditions (buffer, pH, temperature...) to simultaneously digest DNA with several different restriction enzymes.

- 4. "Find information on an enzyme not present in the lab", searches for information on a specific enzyme not present in the user's laboratory, in particular to see if an equivalent enzyme (isoschizomer) could be available in the lab.

- 5. "Find enzymes corresponding to a sequence" searches for enzymes recognizing a selected restriction site sequence.

- 6. "Display our enzyme lab list" displays the personalized list of enzymes present in one's lab, which can be organized in the "Administration" section.

- 7. "Find enzymes sharing reaction conditions" searches for enzymes whose activities are compatible with the selected reaction conditions (buffer, pH, temperature...).

Each of these 7 functions is presented below.

### "Administration"

This password protected access has been designed for laboratories where restriction enzymes are under one person's responsibility (called the RE administrator). The RE administrator can choose from the more than 3500 enzymes present in REtools to customize a REtools lab list to match enzymes actually present in one's laboratory. This customized lab list can be printed out and subsequently posted on a freezer door. More than 3500 enzymes are recorded in the "AllREnar" file present in the REtools folder. We will update this list twice a year to include any newly described enzymes reported in REbase and/or restriction enzyme company catalogs. The commercial availability of each enzyme (from 19 different companies) is indicated. REtools users can update their AllREnar files by simply redownloading and rerunning the REtools installer.

### "Find an enzyme in our lab list"

This function is used to access all the information available for an enzyme present in one's personalized laboratory enzyme list. In addition to the name and recognition site, optimal digestion temperature and optimal buffers from 3 companies (Invitrogen (BRL), Fermentas (MBI), and New England Biolabs (NEB)) are indicated, as well as the percentage of activities in the other buffers.

More specific characteristics of the selected enzyme are proposed when available:

• heat inactivation conditions when applicable. This is of interest when the researcher wants to kill enzyme activity before performing subsequent reactions in the same tube without having to worry about the first enzyme's activity.

• time, temperature and buffer conditions as recommended by the manufacturers.

• sensitivity to methylation, with each possible methylase action and their corresponding effect on cleavage being specified.

• potential isoschizomers (identical site and cleavage) present within the laboratory enzymes.

• number of flanking nucleotides required for cleavage (for example, useful when designing PCR primers harboring restriction sites at their extremities).

### "Perform multiple digestions"

**Figure 3:**
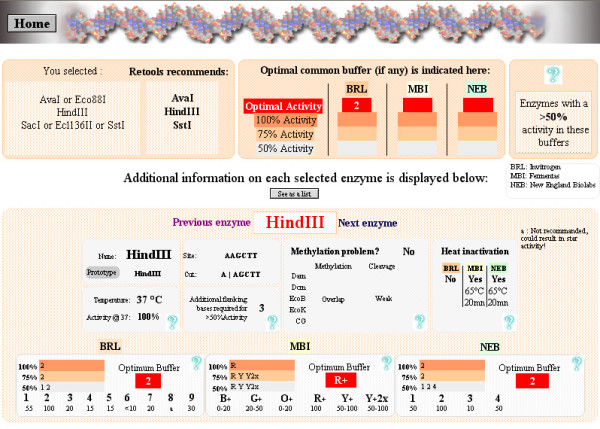
Multiple digest screen. Enzymes selected by the user are indicated, as well as those chosen among them by REtools as the most suited for common conditions. Optimum buffer(s) are indicated, down to a minimum of 50% activity. In addition, information on each enzyme is visible in the lower panel.

This section is of great interest to determine optimum reaction conditions for the chosen enzymes when multiple digestions are performed. The user selects the enzymes to be used for the multiple digestions from the pop up lab list. REtools analyzes the enzymes selected, and additionally proposes isoschizomers (when available) by default. This is important, since there may be cases where two enzymes do not share common reaction conditions, whereas the isoschizomers would. REtools also indicates that neoisoschizomers (identical site, but distinct cleavage) are available, but does not include them in the analysis, except if the user chooses to, since the cleavage pattern is different from the originally selected enzyme. A warning is also displayed in case some of the selected enzymes may be affected by methylation. Once the choice of enzyme has been validated, a screen on which all information related to the multiple digestions is displayed (Fig. 3.).

### "Find information on an enzyme not present in the lab"

This is similar to "Find an enzyme in our lab list", except that all known enzymes are accessible. Importantly, REtools will directly link the searched enzyme to isoschizomers (same site and same cleavage) and neoisoschizomers (same site but distinct cleavage) that may be present in one's personalized laboratory list. This is quite useful, since other enzymes present in one's laboratory may be perfectly compatible with the planned digestion, but unknown to the scientist.

### "Find enzymes corresponding to a sequence"

This section of REtools is designed to determine all the existing restriction enzymes recognizing a specific nucleotide sequence. The prototype enzyme (first discovered restriction enzyme for a specific site and cleavage) for the chosen sequence is listed, as well as all the isoschizomers commercially available. All other prototypes matching the same nucleotide sequence, but with a distinct cleavage pattern, are also displayed. Further inquiries on these prototypes can be made by clicking on their names to find out, for example, if they or an isoschizomer, are present in one's personalized laboratory list. Additionally, REtools indicates the effects that common modifying enzymes, like Klenow or Mung Bean nuclease, would have on the DNA extremities following digestion with the chosen enzyme.

### "Display our enzyme list"

**Figure 4:**
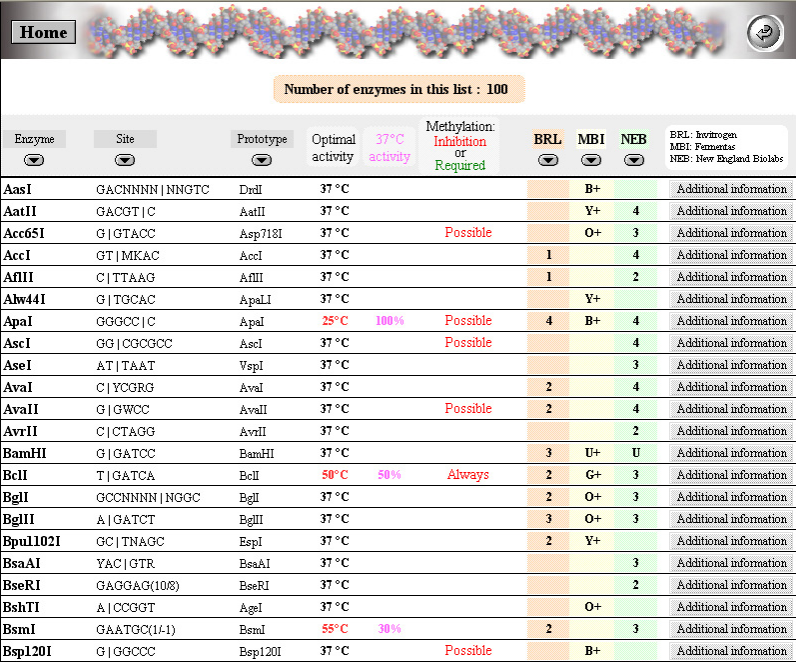
List of the restriction enzymes present in the laboratory.

This is to directly visualize the restriction enzymes available in one's laboratory (Fig. 4.). This list can be sorted according to enzyme name, nucleotide site or prototype name. Recommended digestion temperatures are indicated, as well as the percentage of activity at 37°C if the recommended temperature is different. If applicable, inhibition by or requirement for methylated DNA is mentioned. Finally, recommended buffers are noted for the three major restriction enzyme companies. Further information on any enzyme is accessible directly from this list.

### "Find enzymes sharing reaction conditions"

This function of REtools aims at finding among all known enzymes those which share selected reaction conditions and/or specificities, such as methylation sensitivity. This is of particular interest when an already digested sample must be redigested to separate comigrating fragments. By entering the reaction conditions, the list of compatible enzymes will be displayed, allowing the scientist to select the one(s) that will correspond best to the situation.

## Discussion

Organization is an important part in efficiently running a laboratory. Quite often, the high turnover of students and postdoctoral fellows associated with dynamic laboratories makes tracing and archiving biological samples and reagents quite difficult. In order to reduce these problems, we set up to develop laboratory-oriented applications, based on FileMaker Pro. The originality of these applications is that they were created to organize daily events in the laboratory, rather than forcing users to adapt to the software. The two first applications so far developed and published by our laboratory (FCSM, for the monitoring and organization of samples frozen in liquid nitrogen or in freezers [5], and MICE, for the management of mice projects [6]) obtained a very positive feedback from the scientific community.

In the recent years we became more and more aware of the problems and limitations in restriction enzyme usage. Scientists cannot reasonably keep up with the permanently growing panel of potential alternative solutions when it comes to selecting a restriction enzyme for a particular digestion. We thus looked for a program to assist scientists both in the selection process among enzymes present in the laboratory, as well as in the choice of optimal reaction conditions for the restrictions planned. To our astonishment, very few solutions were available, and most of these were restricted to reagents sold by their manufacturers, such as the previously described NEB's "NEBCutter" [1], and Promega's "Restriction Enzymes Resource" [3]. Another program, RE Finder [7] from Roche, is limited to 339 enzymes and does not help with multiple digestions. None of these three software allow following a personalized laboratory enzyme list. Only one program, EnzymeX [4], developed by molecular biologists in the Netherlands Cancer Institute, responded to most of our needs. This program does not however assist in multiple digestions involving more than two enzymes, nor does it propose alternate iso(neo)schizomers when the selected enzymes are not present in one's customized laboratory list. Finally, EnzymeX exclusively runs on MacOSX 10.2 or later versions, which excludes its use in laboratories running on Windows.

For these reasons, we designed REtools, whose philosophy relies first on the optimization of the laboratory enzyme stock usage, and second on a user-friendly and intuitive program interface, so that people really work with it. REtools provides information on both individual enzyme reaction conditions, and on multiple digest conditions. REtools also allows users to find alternative enzymes that may be present in the laboratory if the enzyme originally chosen is missing, as well as determining all possible enzymes able to recognize a selected restriction site. REtools will make the chore of browsing restriction enzyme catalogs, in the hope of finding the right piece of information, a thing of the past.

REtools can be distributed to all laboratory members since it is provided as a stand-alone application that does not require any third party license to run. To facilitate handling of the enzyme lab stock, an administrator can be designated, who will be in charge of customizing REtools to the enzymes present in the laboratory. The file created (lab list) during this process can then be distributed to all users, who will then work with the actual enzymes present in laboratory.

REtools and its user manual (pdf) [see Additional file 1] are available to the academic community, free of charge, upon request to: pognonec@unice.fr. Please enter: "REtools" in the subject field.

## Conclusion

REtools is a user friendly program for organizing laboratory enzyme stocks. We designed it to make restriction enzyme use and reaction condition determination straightforward and efficient. In particular, we made the usually unexplored potential of isoschizomers accessible to all. Finally, many of the commonly overlooked subtleties of restriction enzyme work are automatically provided by REtools. REtools is a stand alone application, and thus can be distributed to all laboratory members without any requirement for a third party license. To facilitate handling of the enzyme lab stock, an administrator can be designated, who would be in charge of customizing REtools with the enzymes present in one's own laboratory. REtools and its user manual (pdf) are available to the academic community, free of charge, upon request to: pognonec@unice.fr. Please enter: "REtools" in the subject field.

## Availability and requirements

**Project name: **REtools

**Project home page: **

**Operating system(s): **Windows and MAC

**Programming language: **FilemakerPro

**Other requirements: **None

**License: **GNU GPL

**Any restrictions to use by non-academics: **None

## Authors' contributions

The structure of the program was mainly developed by P. Martin, and the interface was mostly the work of P. Pognonec with the help of K.E. Boulukos.

**Figure 5:**
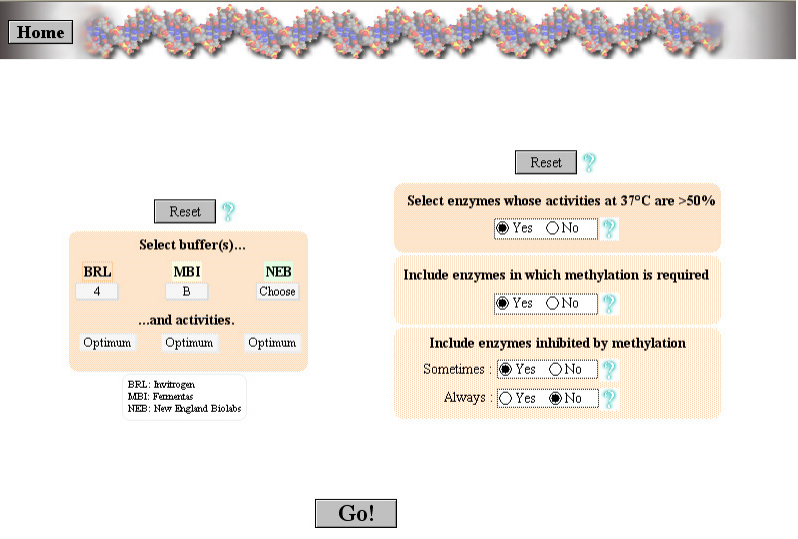
Search of restriction enzymes sharing similar reaction conditions.
